# Surgery for patients with diffuse atherosclerotis disease

**DOI:** 10.1186/1749-8090-8-S1-O3

**Published:** 2013-09-11

**Authors:** Z Mitrev, T Anguseva, I Milev

**Affiliations:** 1 Special Hospital for Surgery Fillip II, Skopje, Macedonia

## Background

An increasing number of patients with peripheral and carotidal vascular disease are undergoing coronary artery bypass grafting. Such patients have an increased risk of adverse outcomes. Our aim was to quantify the effect of on time cardiopulmonary bypass in this group of patients.

## Methods

Between March 2006 and March 2013, 6798 consecutive patients underwent coronary artery bypass grafting; 367 (5.4%) had peripheral and carotidal vascular disease. We used multivariate logistic regression analysis to assess the effect of multimorbidity on post-operative in-hospital mortality and morbidity, while adjusting for treatment selection bias.

## Results

The primary vascular diagnosis was abdominal aortic aneurysm (AAA) in combination with cerebrovascular and peripheral vascular disease in 105 patients (mean age, 67 years), cerebrovascular disease (CVD) in 677 (mean age, 64 years), and lower extremity ischemia (ASO) in 693 (mean age, 61 years), and combination of cerebrovascular and periferial vascular disease in 365 patients (mean age 60 years). All 1840 patients had severe correctable CAD In patients with cerebrovascular, peripheral and combination with coronary artery disease, bypass surgery was performed after resolving of primary vascular disease. In group of patients with AAA, it was operated after resolving a vascular disease, and ate the end abdominal aneurysm was replaces with a graft. The overall operative mortality for 1840 operated patients with cardiac and peripheral vascular procedures was 1.2% (22 pat). Like a postoperative complications stroke incidence was 2.5% (46 pts). Postoperative hospital stay was 5.5 days.

## Conclusion

Planned by-pass surgery is safe in patients with peripheral vascular disease, with acceptable results. The incidence of postoperative stroke is substantially reduced when avoiding cardiopulmonary bypass in patients with present carotidal disease and peripheral vascular disease.

